# Complete Genome Sequence of a *Blochmannia* Endosymbiont of *Colobopsis nipponica*

**DOI:** 10.1128/MRA.01195-20

**Published:** 2021-04-29

**Authors:** Jongsun Park, Hong Xi, Jonghyun Park

**Affiliations:** a InfoBoss, Inc., Seoul, Republic of Korea; b InfoBoss Research Center, Seoul, Republic of Korea; Indiana University, Bloomington

## Abstract

*Blochmannia* endosymbionts (*Gammaproteobacteria*) live in bacteriocytes, which are specialized cells found in the genus *Camponotus* and its neighbor genera. In this announcement, we describe the complete genome sequence of the *Blochmannia* endosymbiont of *Colobopsis nipponica*, which originated from a colony collected in the Republic of Korea.

## ANNOUNCEMENT

Endosymbionts are very commonly identified among at least 15% to 20% of insect species (1), including ants (2) and planthoppers (3). The genus *Camponotus* and its neighbor genera have developed a strong relationship with the *Gammaproteobacteria* genus *Blochmannia*, especially in the early life stage (4). Their roles have been predicted to enrich the nourishment and immunity of the host ants in the bacteriocytes of the ants’ midgut (2, 4, 5). Six *Blochmannia* complete genomes are available (6–9), which can be used to understand species-specific adaptations at the genomic level.

Although large numbers of ant mitochondrial and whole genomes are available because of the rapid development of next-generation sequencing technologies (10–29), one *Colobopsis* complete mitochondrial genome (30) and one endosymbiont bacterial genome (8) are available. More genomes of endosymbionts in this genus can provide their detailed characteristics.

A colony of *Colobopsis nipponica* was collected in a coastal forest of Geoje Island, Republic of Korea (34°51′59.6″N, 128°44′22.0″E) (number KFDS00186; InfoBoss Cyber Herbarium). Total DNA was extracted from multiple *C. nipponica* workers by using DNeasy blood and tissue kits (Qiagen, Hilden, Germany). The sequencing library was constructed using the TruSeq Nano DNA library preparation kit (Illumina, San Diego, CA) following the manufacturer’s recommendations, with ∼350-bp DNA fragments. Genome sequencing was performed using a HiSeq X system at Macrogen, Inc. (Republic of Korea), with extracted DNA from *Colobopsis nipponica*, yielding 38.41 million 151-bp reads. Bioinformatic analyses were performed with default parameters except where otherwise noted. *De novo* assembly was conducted by Velvet v1.2.10 (31) with a minimum depth of 30×, after raw read filtering with Trimmomatic v0.33 (32). Contig sequences were selected based on BLASTN results against the nonredundant data set; matched sequences were from endosymbiont bacteria with >95% query coverage and >80% identity. GapCloser v1.12 (33), BWA v0.7.17 (34), SAMtools v1.9 (35), and Geneious R11 v11.1.5 (Biomatters Ltd., Auckland, New Zealand) were used for filling gaps and conforming assembled sequences. Assembled genome sequences were confirmed as a circular genome via overlapping 2-kb sequences on both ends. After completion of the genome, all bases were confirmed using BWA v0.7.17 (34) and SAMtools v1.9 (35). Genome annotation was conducted by the NCBI Prokaryotic Genome Annotation Pipeline (PGAP) (36).

The complete genome of the *Blochmannia* endosymbiont of *Colobopsis nipponica* was 728,116 bp long, its coverage was 232.44×, and its GC content was 28.7%. It is the second shortest genome, after that of “*Candidatus* Blochmannia vafer” (GenBank accession number CP002189), which was isolated from *Camponotus vafer* (8). The genome of the *Blochmannia* endosymbiont of *Colobopsis obliquus* (GenBank accession number CP010049) (773,940 bp) (8) presents no inversion against our genome. A total of 609 protein-coding genes (PCGs), 40 tRNAs, 3 rRNAs, 2 noncoding RNAs, and 1 transfer-messenger RNA were predicted. The number of PCGs is the third highest among all available *Blochmannia* complete genomes, after those of the *Blochmannia* endosymbionts of *Camponotus nipponensis* (9) and *Camponotus pennsylvanicus* (6). The number of tRNAs is the same as those of the aforementioned two genomes, the highest among seven genomes.

We expect that our genome sequence will be used for comparative genomic analyses of ant endosymbionts to clarify their genomic characteristics, as well as their coevolutionary histories with host species.

### Data availability.

The whole-genome sequencing project for the *Blochmannia* endosymbiont of *Colobopsis nipponica* was deposited in DDBJ/ENA/GenBank under accession number CP046533, BioProject number PRJNA592762, and BioSample number SAMN13439370. Raw sequences were deposited under SRA accession number SRR12768939.
